# Comparison of Clinical Outcomes Between Ticagrelor and Clopidogrel in Acute Coronary Syndrome: A Comprehensive Meta-Analysis

**DOI:** 10.3389/fcvm.2021.818215

**Published:** 2022-01-27

**Authors:** Mengyi Sun, Weichen Cui, Linping Li

**Affiliations:** ^1^Department of Clinical Laboratory, Jining Academy of Medical Sciences, Jining, China; ^2^Department of Clinical Laboratory, Jiaxiang Women and Children's Hospital, Jining, China; ^3^Department of Cardiology, Jining Academy of Medical Sciences, Jining, China

**Keywords:** ticagrelor, clopidogrel, acute coronary syndrome, percutaneous coronary intervention, meta-analysis

## Abstract

**Background:**

Ticagrelor is currently recommended for patients with the acute coronary syndrome (ACS). However, recent studies have yielded controversial results.

**Objective:**

To compare the clinical outcomes between ticagrelor and clopidogrel in patients with ACS.

**Methods:**

Three electronic databases were queried until April 25, 2021. We defined major adverse cardiovascular events (MACEs) as the primary efficacy endpoint. The secondary efficacy endpoints included stroke, stent thrombosis, cardiovascular death, all-cause death, and myocardial infarction. The safety endpoints were (major and minor) bleeding. Odds ratios (ORs) and 95% CIs were calculated to represent the estimated effect sizes.

**Results:**

A total of 270,937 patients with ACS from 10 clinical trials and 18 observational studies were included. No significant difference was detected in MACE (OR 0.81, 95% CI 0.60–1.08, *p* = 0.15, *I*^2^ = 64.83%). However, ticagrelor introduced a higher risk of bleeding (1.46, 1.17–1.83, 0.00, 61.66%) and minor bleeding (1.71, 1.33–2.21, 0.00, 4.65%) in clinical trials. The results of secondary efficacy endpoints differed in the clinical trials and observational studies. Subgroup analysis demonstrated that ticagrelor showed better therapeutic effects in patients who underwent the percutaneous coronary intervention (PCI) (0.38, 0.23–0.63, 0.00, 0) than those intended for PCI (1.03, 0.76–1.38, 0.87, 64.26%). Meanwhile, ticagrelor showed different therapeutic effects on patients with ACS of different ethnicities and different countries.

**Conclusion:**

This meta-analysis demonstrated that ticagrelor is not superior to clopidogrel in MACE but is associated with a higher risk of bleeding in patients with ACS. Different PCI strategies, ethnicities, and countries may be the factors that contribute to different therapeutic effects of ticagrelor.

**Systematic Review Registration:**

This study is registered with PROSPERO (CRD42021251212).

## Introduction

Currently, cardiovascular disease (CVD) is the largest contributor to the disease burden, accounting for approximately one-third of the global deaths (1). Besides, acute coronary syndrome (ACS), as a common and serious CVD, has a dramatically increased incidence with age, proposing a great challenge to public medical care. Dual antiplatelet therapy (DAPT) is the mainstay treatment strategy for ACS, with timely vascularization as needed (2, 3). As for the choice of antiplatelet agent, ticagrelor is recommended over clopidogrel for patients with ACS or who have received the percutaneous coronary intervention (PCI) in the 2016 American College of Cardiology (ACC)/American Heart Association (AHA) guidelines and the 2018 European Society of Cardiology (ESC)/European Association for Cardio-Thoracic Surgery (EACTS) guidelines (2, 4).

As a novel adenosine diphosphate receptor antagonist, ticagrelor provides faster, more potent, and more stable platelet inhibition than clopidogrel (5, 6). The large Platelet Inhibition and Patient Outcomes (PLATO) trial exhibited that compared with clopidogrel, ticagrelor reduced the incidences of stroke, myocardial infarction (MI), and cardiovascular (CV) death, without elevating the risk of major bleeding (7). However, other large clinical trials (8, 9) and observational studies (10–12) drew controversial conclusions. Meanwhile, several meta-analyses published recently also reported inconsistent results (13–16). Therefore, we conducted a meta-analysis to review previous relevant studies and compared the clinical benefits of ticagrelor and clopidogrel in the ACS population using aspirin to address these conflicting conclusions.

## Methods

This meta-analysis was conducted following the Preferred Reporting Items for Systematic Reviews and Meta-Analyses (PRISMA) guideline (17) (Supplementary Table S1) and has been registered in an international prospective register of systematic reviews PROSPERO (ID: CRD42021251212).

### Literature Search

Three electronic databases, Cochrane, EMBASE, and PubMed library, were searched for eligible citations before April 25, 2021. The following keywords were applied: “ticagrelor,” “clopidogrel,” “myocardial ischemia,” “ACS,” “percutaneous coronary intervention,” and “PCI.” The detailed search strategy is shown in Supplementary Table S2. In addition, references in relevant meta-analyses were manually searched for potential eligibility.

### Inclusion and Exclusion Criteria

We reviewed the full texts of these potentially eligible kinds of literature to determine whether they fulfilled the following inclusion criteria: (1) adult (≥18 years old) patients with ACS who underwent PCI (PCI strategy proportion equal to 100%) or intended for PCI (proportion less than 100%), which were described in titles and abstracts or baseline characteristics forms; (2) clinical trials and observational studies comparing ticagrelor vs. clopidogrel in the context of aspirin use; (3) one or more of the following outcomes reported during any follow-up period: MACE, all-cause death, CV death, MI, stroke, stent thrombosis (ST), and (major or minor) bleeding. Exclusion criteria were as follows: (1) studies with incomplete data, or observational studies with unadjusted endpoints; (2) studies from the same sample source; (3) studies not in English.

### Study Endpoints

The primary efficacy endpoints were trial-defined primary MACEs or efficacy endpoints (described as death/CV death, MI, and/or stoke) (Supplementary Table S4). The secondary endpoints included stroke, ST, MI, CV death, and all-cause death. The safety endpoints were trial-defined bleeding (described as bleeding, major or minor bleeding) (Supplementary Table S4). For the definitions of safety outcomes, if not otherwise specified, we prioritized PLATO definitions when available (Supplementary Table S5).

### Data Extraction

Data from the included citations were independently populated with a standardized data extraction by two researchers, with any discrepancy resolved by a third researcher. The data included the last names of authors, study type, country, publication year, journal, disease subtype, sample size, age, gender distribution, the PCI strategy, dosing regimen, reported outcomes about efficacy and safety, and follow-up duration.

### Quality Assessment

We assessed the bias risk of randomized controlled trials (RCTs) using the Cochrane Collaboration risk-of-bias-tool (RoB 2) (18), which covers five domains of bias to classify the RCTs into three levels (low risk of bias, some concerns, and high risk of bias) (Supplementary Table S6). Meanwhile, we assessed the quality of observational studies using the Newcastle–Ottawa Scale (NOS), which includes eight items for three aspects (selection, comparability, and outcome) assessments (Supplementary Table S7). The NOS adopts the semiquantitative principle of star allocation to assess the literature quality, with a full score of nine stars (19).

### Statistical Analysis

We used the odds ratios (ORs) and 95% CIs to represent the estimated effect sizes, which were obtained *via* the Stata 16.0 software (StataCorp, CollegeStation, TX, USA) (20). Given the inclusion of heterogeneous populations, we chose the random-effects model to pool the effect sizes for this meta-analysis. Furthermore, we used the Higgins' *I*^2^ statistics and Cochran's *Q*-test to estimate heterogeneity across studies. A *p*-value < 0.05 was considered statistically significant.

All the analyses were performed by analyzing data from clinical trials and observational studies separately to reduce heterogeneity caused by different study types. Meanwhile, subgroup analyses were performed to search for potential sources of heterogeneity. In brief, according to different data types of the observational studies, we performed two subgroup analyses, namely, propensity score-matched/adjusted analyses (PA group) and multivariate-adjusted analyses (MA group). In addition, we conducted prespecified subgroup analyses on the included RCTs based on PCI strategy, ethnicity, country, duration of follow-up, and enrollment time.

The stability of our findings was evaluated by sensitivity analyses, which means to calculate the effects by including high-quality RCTs and by only including studies with clinical event committee-adjudicated events in RCTs (21). When more than ten studies were included, the presence of publication bias was investigated by Egger's test and displayed by visual estimation (symmetry) of funnel plots.

## Results

### Eligible Studies and Patient Characteristics

**Figure 1:**
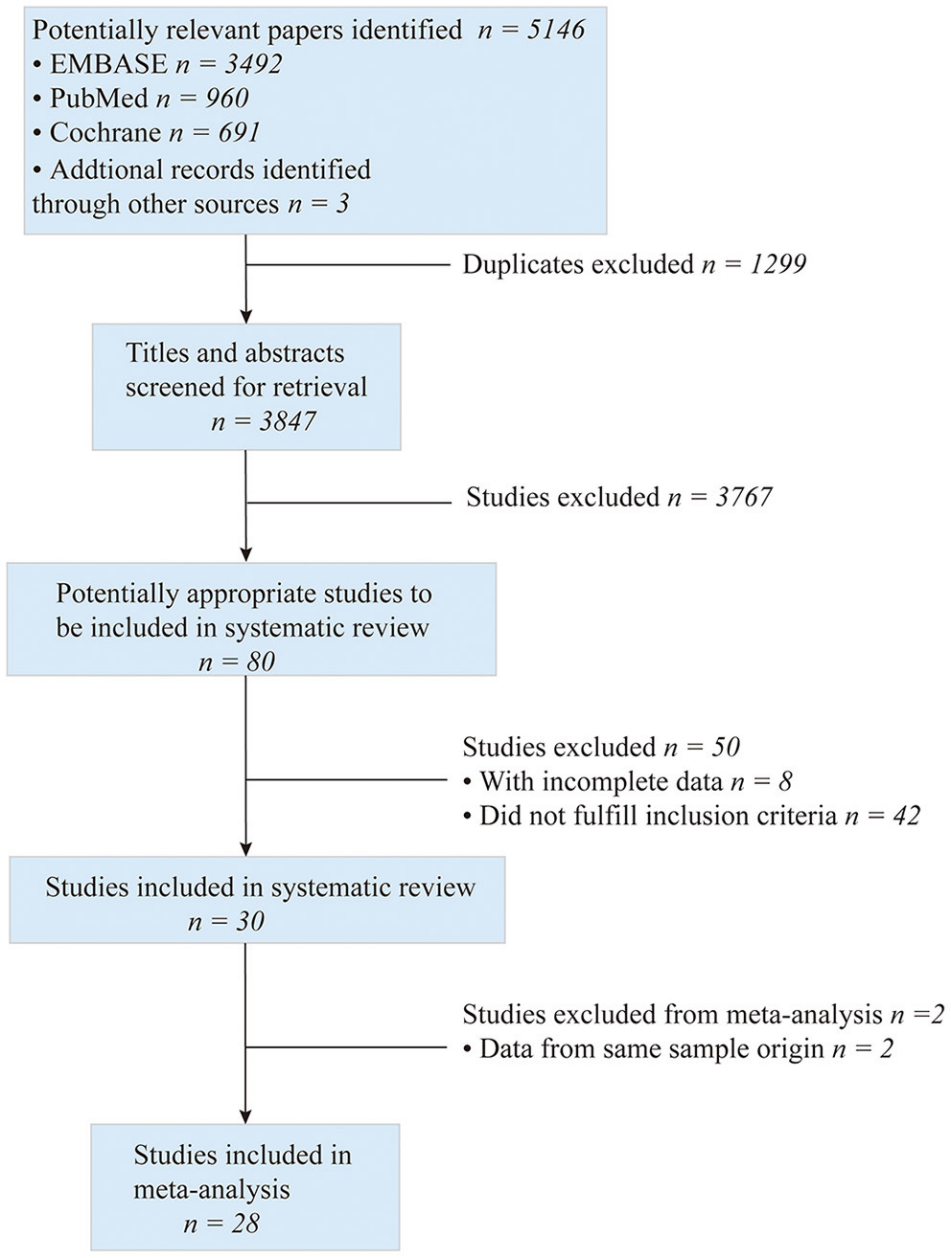
Flowchart diagram of searching and screening of studies.

The process of searching, retrieving, and screening for this meta-analysis is shown in Figure 1. A total of 5,146 potentially relevant kinds of literature were screened. Then 1,299 duplicates were excluded, and 3,767 were retrieved for the title and abstract screening. Subsequently, the full texts of 80 articles were reviewed for eligibility. Among them, 42 articles did not fulfill the inclusion criteria, 8 presented incomplete data, and 2 employed the same cohorts included in our study. Finally, 28 studies with 270,937 patients (88,490 and 182,447 in the ticagrelor and clopidogrel groups, respectively) were included for the meta-analysis. There were 10 clinical trials (5, 7–9, 22–27) and 18 observational studies (10–12, 28–42). The patients were enrolled from 2003 to 2019, and the articles were published from 2007 to 2020. Among them, 19 studies included patients with ACS who underwent PCI, while 9 included those intended for PCI. A total of 254,450 patients received PCI. The countries, in which these studies were conducted, included East Asian countries, such as China, Korea, and Japan, and European and American countries, such as the United States, Canada, Sweden, the Netherlands, and England. In addition, the ethnicities of the cohorts were East Asians and Caucasians. And the duration of follow-up ranged from 30 to 468 days. The main studies and population characteristics are summarized in Supplementary Tables S3, S4.

### Efficacy Endpoints

**Figure 2:**
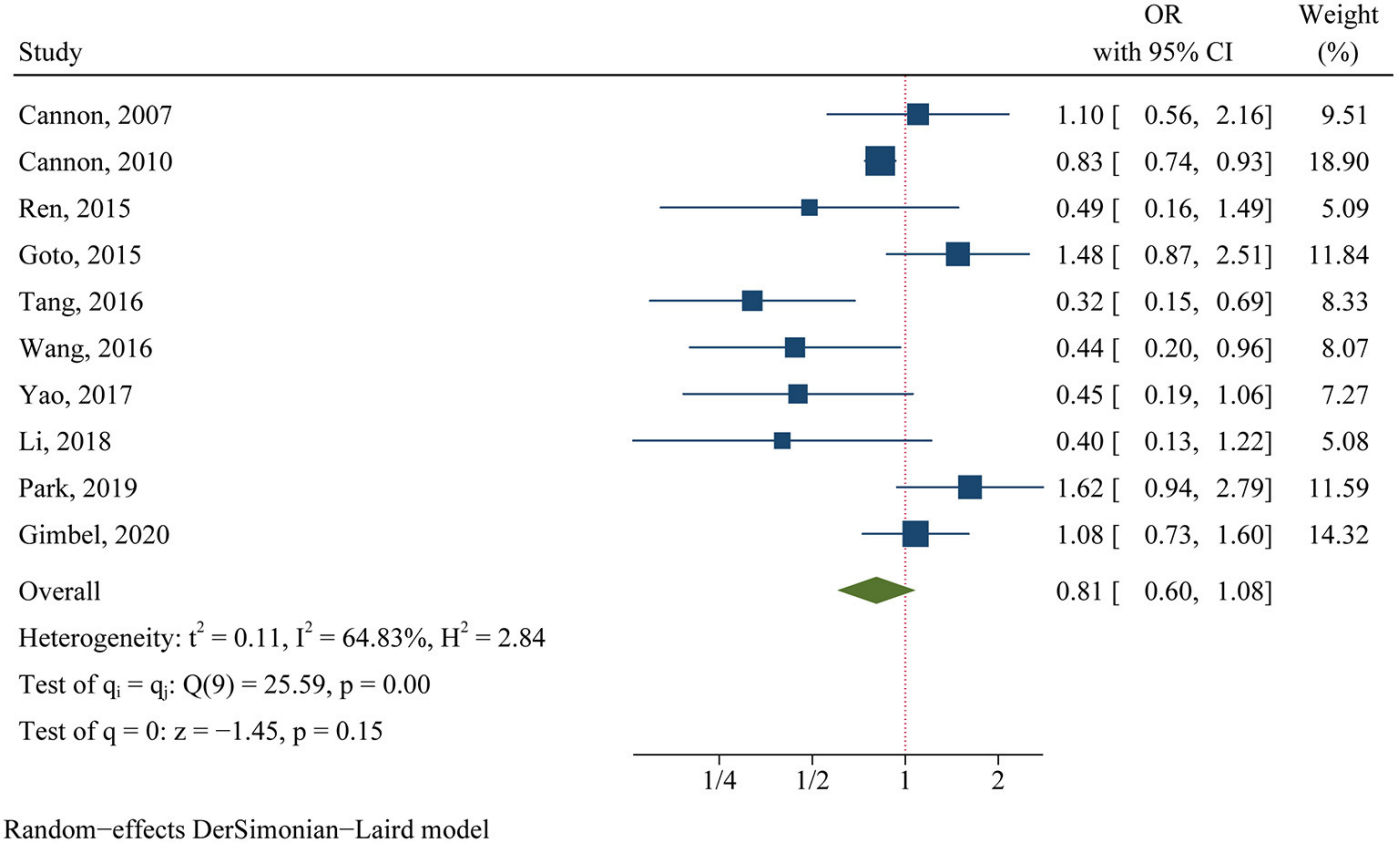
Comparison of the primary efficacy outcomes (MACE) between ticagrelor and clopidogrel treatment in clinical trials.

**Figure 3:**
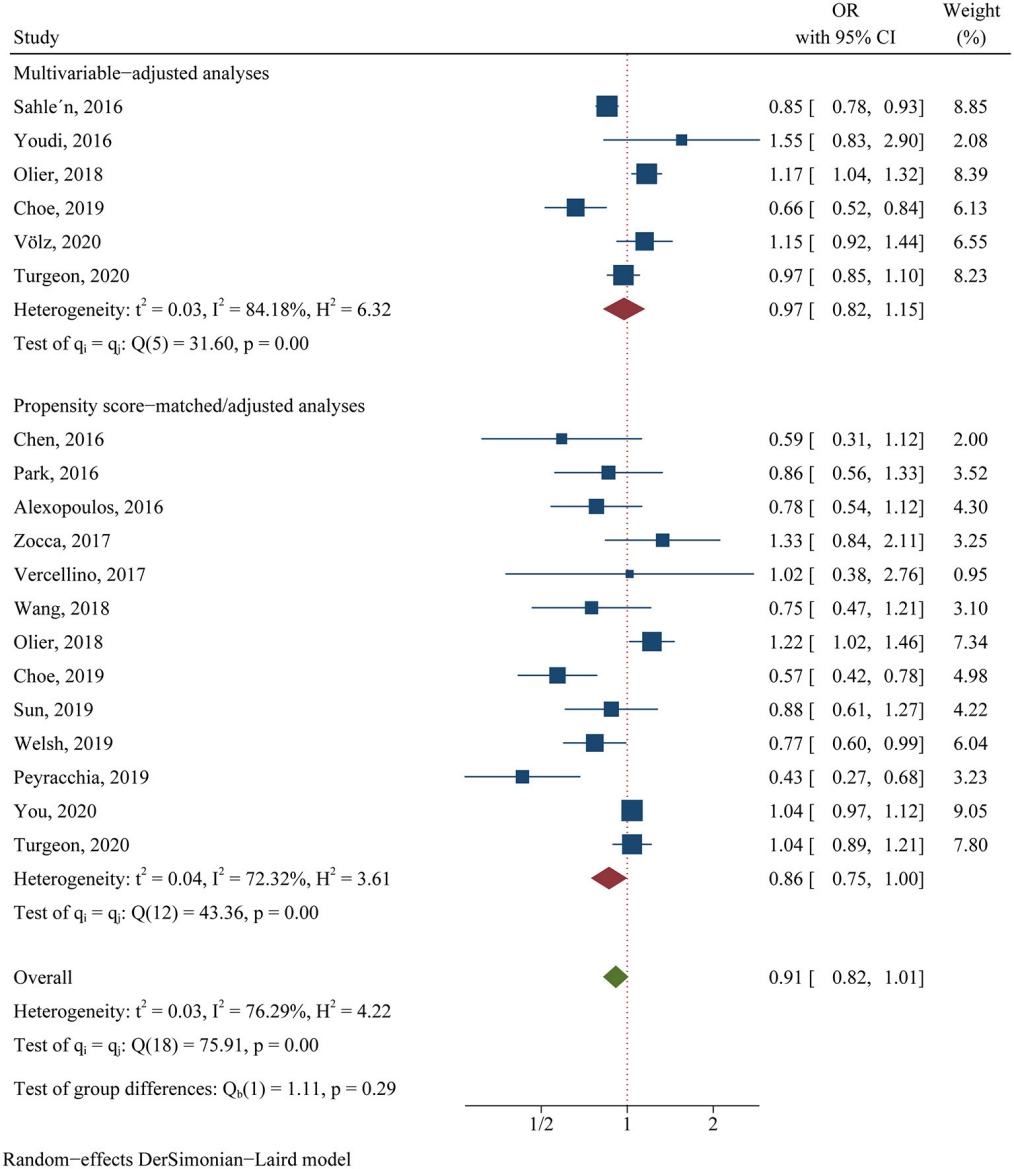
Comparison of the primary efficacy outcomes (MACE) between ticagrelor and clopidogrel treatment in observational studies.

For the clinical trials, no significant difference was found in the primary efficacy endpoint (MACE) between ticagrelor and clopidogrel groups (OR 0.81, 95% CI 0.60–1.08, *p* = 0.15, *I*^2^ = 64.83%; Figure 2). For the observational studies, similar results were obtained in both the MA group (OR 0.97, 95% CI 0.82–1.15, *p* = 0.76, *I*^2^ = 84.18%; Figure 3) and the PA group (OR 0.86, 95% CI 0.75–1.00, *p* = 0.05, *I*^2^ = 72.32%; Figure 3).

Compared with the clopidogrel group, the ticagrelor group demonstrated a reduction in secondary endpoints, including ST (OR 0.72, 95% CI 0.58–0.90, *p* = 0.00, *I*^2^ = 0.00%; Table 1) in clinical trials, all-cause death (OR 0.83, 95% CI 0.70–0.98 *p* = 0.03, *I*^2^ = 69.89%) and CV death (OR 0.66, 95% CI 0.44–0.99, *p* = 0.04, *I*^2^ = 70.59%) in the PA group, and CV death (OR 0.59, 95% CI 0.45–0.79, *p* < 0.001) in the MA group (Table 2).

**Table 1:** Comparison of ticagrelor and clopidogrel treatment for the safety and second efficacy endpoints in clinical trials.

**Outcomes**	**Trials**	**OR (95%CI)**	**I^**2**^**	***p* Value**
Bleeding	8	1.46 (1.17, 1.83)	61.66	0.00 ^*^
Major bleeding	7	1.22 (0.93, 1.61)	49.05	0.14
Minor bleeding	6	1.71 (1.33, 2.21)	4.65	0.00^*^
All-cause death	8	0.85 (0.71, 1.01)	4.05	0.07
CV death	8	0.87 (0.63, 1.22)	30.86	0.43
MI	8	0.87 (0.66, 1.16)	39.12	0.36
Stroke	8	1.08 (0.78, 1.49)	4.41	0.64
Stent thrombosis	3	0.72 (0.58, 0.90)	0.00	0 00^*^

* *significant p-value compared with clopidogrel group. CV death, cardiovascular death; MI, myocardial infarction*.

**Table 2:** Comparison of ticagrelor and clopidogrel treatment for the safety and second efficacy endpoints in observational studies.

**Outcomes**	**PA group**				**MA group**			
	**Studies**	**OR (95%CI)**	***I* ^2^**	***p* Value**	**Studies**	**OR (95%CI)**	***I* ^2^**	***p* Value**
Bleeding	7	1.39 (1.06, 1.83)	76.11	0.02^*^	6	1.15 (0.86, 1.53)	81.88	0.35
Major bleeding	8	1.26 (0.90, 1.75)	75.23	0.17	2	1.12 (0.86, 1.45)	0.00	0.39
Minor bleeding	4	1.61 (1.37, 1.89)	0.00	0.00 ^*^	1	1.21 (1.14, 1.72)	-	0.007^*^
All-cause death	13	0.83 (0.70, 0.98)	69.89	0.03^*^	8	0.93 (0.78, 1.10)	79.69	0.38
CV death	6	0.66 (0.44, 0.99)	70.59	0.04^*^	1	0.59 (0.45, 0.79)	-	<0.001^*^
MI	11	0.99 (0.84, 1.15)	48.41	0.87	4	0.90 (0.71, 1.15)	79.13	0.39
Stroke	11	0.84 (0.65, 1.09)	39.97	0.19	4	0.79 (0.59, 1.06)	52.70	0.12
Stent thrombosis	5	1.18 (0.81, 1.72)	6.26	0.39	2	1.45 (0.89, 2.37)	0,00	0.14

* *significant p value compared with clopidogrel group. CV death, cardiovascular death; MI, myocardial infarction; PA group, propensity score-matched/adjusted analyses; MA group, multivariable-adjusted analyses*.

### Safety Endpoints

Ticagrelor led to significantly higher risks of bleeding (OR 1.46, 95% CI 1.17–1.83, *p* = 0.00, *I*^2^ = 61.66%) and minor bleeding (OR 1.71, 95% CI 1.33–2.21, *p* = 0.00, *I*^2^ = 4.65%) over clopidogrel in clinical trials (Table 1). The increased risks of bleeding (OR 1.39, 95% CI 1.06–1.83, *p* = 0.02, *I*^2^ = 76.11%) and minor bleeding (OR 1.61, 95% CI 1.37–1.89, *p* = 0.00, *I*^2^ = 0.00%) were also identified in the PA group of observational studies (Table 2). However, only the minor bleeding risk (OR 1.21, 95% CI 1.14–1.72, *p* = 0.007; Table 2) increased significantly in the MA group of observational studies.

### Subgroup Analysis

In the subgroup of PCI strategy, patients underwent PCI (OR 0.38, 95% CI 0.23–0.63, *p* = 0.00, *I*^2^ = 0) benefited more from ticagrelor than those intended for PCI (OR 1.03, 95% CI 0.76–1.38, *p* = 0.87, *I*^2^ = 64.26%) regarding MACE (Table 3). However, ticagrelor introduced a higher risk of bleeding in the patients either underwent PCI (OR 1.64, 95% CI 1.07–2.51, *p* = 0.02, *I*^2^ = 0) or intended for PCI (OR 1.44 95% CI 1.11–1.86, *p* = 0.01, *I*^2^ = 68.56%; Table 3).

**Table 3:** Subgroup comparison for MACE and bleeding outcomes between ticagrelor and clopidogrel treatment in RCTs.

**Outcomes**	**Subgroup**		**Trials**	**Participants**		**OR (95% CI)**	**I^**2**^**	***p* Value**
				**Ticagrelor**	**Clopidogrel**			
MACE	PCI strategy							
		Intended for PCI	6	8,469	8,403	1.03 (0.76, 1.38)	64.26	0.87
		Underwent PCI	3	421	541	0.38 (0.23, 0.63)	0.00	0.00^*^
	Ethnicity							
		East Asian	6	1,322	1,441	0.67 (0.36, 1.25)	77.78	0.21
		Caucasian	2	7,066	7,003	0.84 (0.75, 0.94)	0.00	0.00^*^
	Asian countries	China	4	521	641	0.40 (0.26, 0.60)	0.00	0.00 ^*^
		Korea	1	400	400	1.62 (0.94, 2.79)	-	0.07
		Japan^#^	1	401	400	1.48 (0.87, 2.51)	-	0.15
	Follow-up duration	< =1	1	334	327	1.05 (0.52, 2.52)	-	0.71
		>1	9	8,890	8,944	0.83 (0.61, 1.12)	67.54	0.22
		< =6	4	928	914	0.66 (0.35, 1.26)	63.85	0.21
		>6	6	8,296	8,357	0.96 (0.69, 1.33)	67.66	0.79
Bleeding	PCI strategy							
		Intended for PCI	6	8,469	8,403	1.44 (1.11, 1.86)	68.56	0.01^*^
		Underwent PCI	2	361	481	1.64 (1.07, 2.51)	0.00	0.02^*^
	Ethnicity							
		East Asian	5	1,262	1,381	1.81 (1.43, 2.29)	0.00	0.00^*^
		Caucasian	2	7,066	7,003	1.09 (0.99, 1.20)	0.00	0.07
	Asian countries	China	3	461	581	1.64 (1.13, 2.37)	0.00	0.01^*^
		Korea	1	400	400	2.29 (1.34, 3.92)	-	0.02^*^
		Japan^#^	1	401	400	1.80 (0.87, 2.51)	-	0.001^*^
	Follow-up duration							
		< =1	1	334	327	1.23 (0.71, 2.12)	0.00	0.62
		>1	8	8,830	8,884	1.46 (1.17, 1.83)	61.66	0.00^*^
		< =6	3	868	854	1.23 (0.88, 1.72)	0.00	0.22
		>6	6	8,296	8,357	1.54 (1.17, 2.02)	71.98	0.00^*^

# *Data comes from Goto 2015, in which Japanese proportion was 90%;*

* *significant p value compared with clopidogrel group. MACE, major adverse cardiovascular event; RCT, randomized controlled trials; PCI, percutaneous coronary intervention*.

Subgroup analysis based on different ethnicities was performed to compare the clinical outcomes of ticagrelor and clopidogrel in Caucasian and East Asian populations, respectively. Ticagrelor showed a superior MACE reducing effect (OR 0.84, 95% CI 0.75–0.94, *p* = 0.00, *I*^2^ = 0; Table 3) and a lower risk of bleeding (OR 1.09, 95% CI 0.99–1.20, *p* = 0.07, *I*^2^ = 0; Table 3) over clopidogrel in Caucasian patients. However, the results were inconsistent in East Asian populations. Ticagrelor was comparable with clopidogrel regarding MACE (OR 0.67, 95% CI 0.36–1.25, *p* = 0.21, *I*^2^ = 77.78%; Table 3) and introduced a higher bleeding risk (OR 1.81, 95% CI 1.43–2.29, *p* = 0.00, *I*^2^ = 0; Table 3).

Subgroup analysis based on different Asian countries showed that Chinese patients benefited more from ticagrelor than those in Korean and Japanese, while the bleeding risk of ticagrelor significantly increased in all three Asian countries (Table 3). Further subgroup analysis was conducted to analyze the safety and efficacy of ticagrelor and clopidogrel based on different follow-up duration. It showed MACE was comparable between the two groups during the follow-up duration, while the bleeding risk of both two groups increased with a longer follow-up duration (Table 3).

Ticagrelor became widely available in 2012 (43). We made a subgroup analysis about the efficacy and safety of ticagrelor to detect the time effect. There was a lower MACE incidence before 2012, mainly relying on the lower incidences of CV death, MI, and ST under ticagrelor treatment while the bleeding risk was acceptable. After 2012, both the incidence of MACE and the second efficacy endpoints were comparable between the two groups, but the risk of bleeding and minor bleeding was higher than clopidogrel (Supplementary Table S8).

### Sensitivity Analyses and Publication Bias

Sensitivity analyses were performed by including high-quality RCTs and by only including studies with clinical event committee-adjudicated events in RCTs. The results remained consistent (Supplementary Tables S9, S10).

**Figure 4:**
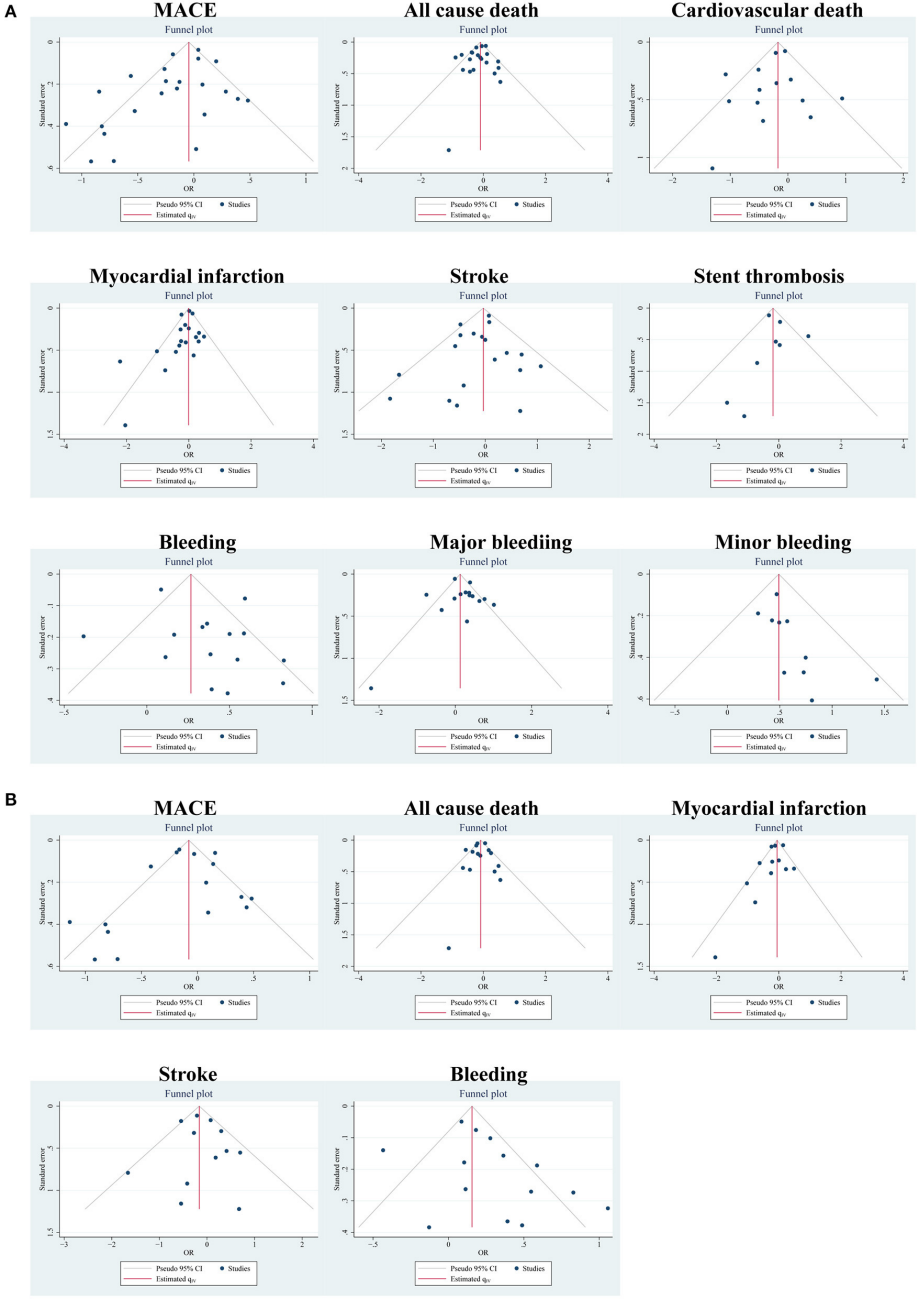
Publication bias of outcomes using funnel plots: **(A)** in the PA group and clinical trials; **(B)** in the MA group and clinical trials.

According to different data types, publication bias was investigated in the two pairings: the PA group and clinical trials, and the MA group and clinical trials. By funnel plots (Figure 4) and Egger's test (Table 4), we detected publication only in MACE and MI between the PA group and clinical trials. The results of the non-parametric trim-and-fill analysis showed that five studies were filled for MACE with the total results influenced, and two were filled for MI with the total results unaffected (Supplementary Table S11).

**Table 4:** Publication bias of outcomes using the Egger's test.

**Outcomes**	**PA group and clinical trials**		**MA group and clinical trials**	
	**Studies**	**Prob > |z|**	**Studies**	**Prob > |z|**
MACE	23	0.0197^*^	16	0.1163
All-cause death	21	0.9956	16	0.7295
CV death	14	0.7616	9	NA
MI	20	0.0486^*^	13	0.1195
Stoke	19	0.7060	12	0.7660
Stent thrombosis	10	0.5747	7	NA
Bleeding	15	0.3814	14	0.0718
Major bleeding	15	0.4940	9	NA
Minor bleeding	10	0.1490	7	NA

* *Publication bias was detected in this outcome. MACE, major adverse cardiac events; CV death, cardiovascular death; MI, myocardial infarction; NA, not available; PA group, propensity score-matched/adjusted analyses; MA group, multivariable-adjusted analyses*.

## Discussion

This meta-analysis, based on 28 studies, suggested that ticagrelor was not only inferior to clopidogrel in patients with ACS but also related to an increased bleeding risk, whereas ticagrelor was more effective for the patients who underwent PCI than clopidogrel, as it significantly reduced the incidence of MACE. Meanwhile, this meta-analysis revealed that Caucasians and East Asians had inconsistent safety and efficacy profiles under ticagrelor treatment. Among East Asian patients, Chinese benefited more from ticagrelor than Korean and Japanese. Care should, therefore, be taken to screen the eligible population when applying ticagrelor and the issue of increased bleeding risk with longer follow-up duration under ticagrelor treatment was of concern.

Recently, several studies have revisited the issues concerning clinical applications of ticagrelor and clopidogrel. These meta-analyses got no consensus on the efficacy and safety of ticagrelor treatment. They either mixed all studies together and ignored the heterogeneity between clinical trials and observational studies, or did not perform further subgroup analyses based on other studies (or population) characteristics (13, 15, 16). In this meta-analysis, we performed prespecified subgroup analyses for MACE and bleeding in RCTs according to PCI strategy, ethnicity, country, and follow-up duration. Additionally, we performed two subgroup analyses (the PA and MA groups) in observational studies according to different data types. We conducted a comprehensive analysis from both clinical trial and real-world practice considerations to compare the clinical outcomes of ticagrelor and clopidogrel in different subgroups. Therefore, some definitive evidence can be provided for clinicians to choose between ticagrelor and clopidogrel.

We found similar primary efficacy results of ticagrelor treatment in both clinical trials and observational studies, though the result of the MA group differed slightly. The results of the secondary efficacy endpoints differed in clinical trials and observational studies, which can be attributed to inherent differences between study types. In brief, clinical trials (especially RCTs) match baseline characteristics well. However, although clinical factors associated with treatment selection in observational studies can be matched by propensity score-matched/adjusted analyses or multivariable-adjusted analyses, there are still some unadjusted or incomplete adjustment variables that may affect the results. This may explain the inconsistent results of bleeding in the MA group and the secondary endpoints in clinical studies and observational studies.

In addition to pharmacological treatment with DAPT, the primary management of patients with ACS involves early invasive strategies, namely, coronary angiography, PCI, and even coronary artery bypass grafting (CABG) (2, 4, 44). Considering that many high-risk patients with ACS received PCI after coronary angiography, we included those who underwent or intended for PCI to better compare the clinical outcomes. Subgroup comparison in this study revealed that ticagrelor could significantly reduce the incidence of MACE in patients who underwent PCI compared with those intended for PCI. Patients with ACS who received PCI or CABG had different clinical characteristics from those who received medical treatment alone. Platelet activation plays a significant role in PCI-related thrombotic events. Intra-individual variability in platelet reactivity occurred in patients undergoing elective coronary stenting (45). In addition, high clopidogrel on-treatment platelet reactivity (HTPR) enhanced the occurrence of ischemic adverse events (46). On the contrary, ticagrelor produces a more potent antiplatelet effect than clopidogrel. Bonello et al. reported that ticagrelor induced an optimal platelet reactivity inhibition in patients who underwent PCI (47), and it provided an approach for overcoming HTPR (48). Patients who underwent PCI benefit more from ticagrelor treatment than those intended for PCI, providing valuable suggestions for clinicians to choose between ticagrelor and clopidogrel according to the PCI strategy.

In terms of ethnicity, Caucasians made up 91% of the PLATO trial population, whereas East Asians accounted for only 6%. Caucasians and East Asians differ substantially in phenotypes and genomics. Therefore, recommendations based on Caucasians do not necessarily apply to East Asians (13). We conducted subgroup analyses in Caucasians and East Asians, respectively. The results were different between the two subgroups. East Asians are thought to be more prone to bleeding events than Caucasians but are relatively resistant to adverse ischemic outcomes after PCI (the so-called “East Asian paradox”) (49–51). In addition, cytochrome P450 2C19 loss-of-function alleles associated with high platelet reactivity are more common in Asian populations (52). In a nutshell, there are significant ethnic differences between Caucasian and East Asian patients in terms of thrombosis, platelet P2Y12 receptor inhibition, and predisposition to bleeding complications (49). Those differences might partly contribute to this interpopulation disparity. Considering the difference between ischemia and bleeding risk, the choice of antithrombotic drugs may differ between East Asian patients and Caucasian patients. The unique risk-benefit trade-off in East Asian patients was worthy of attention. The number of RCTs included in this meta-analysis is relatively small, and more RCTs are required for further elaboration.

In East Asia, the MACE incidence was significantly reduced by ticagrelor in Chinese compared with Korean and Japanese, which may be partially attributed to the different proportions of patients who underwent PCI. Specifically, 81.5% of the patients with ACS in the Korean TICAKOREA trial (8) and 84.8% of the patients with ACS in the Japanese PHILO trial (9) were assigned to the ticagrelor group underwent PCI. More importantly, data from four Chinese trials showed that up to 94% of the patients with ACS in the ticagrelor group underwent PCI. Additionally, the baseline characteristics, diagnostic criteria, and dosage regimen might be the reasons for the differences. The efficacy of ticagrelor in Asian populations needs to be validated by larger trials.

In this meta-analysis, the primary endpoint of MACE was comparable between ticagrelor and clopidogrel in the patients with ACS. However, ticagrelor introduced an increased bleeding risk with the extension of follow-up duration, which deserves attention. Researchers have explored the possibility of a shorter-duration DAPT. Abbreviated-duration ( ≤ 6 months) DAPT in the CAD population did not significantly increase the incidence of MACE, but dramatically reduced the risk of major bleeding (53). Additionally, the safety and efficacy of short-duration ( ≤ 3 months) DAPT in elderly patients were also acceptable (54). However, the optimal DAPT duration after PCI still remains a topic of emerging interest. There are growing concerns regarding P2Y12 inhibitor monotherapy. Valgimigli et al. proved that, compared with DAPT, P2Y12 inhibitor monotherapy was non-inferior in efficacy endpoints, and with a reduced risk of major bleeding (55). Considering the reduced bleeding risk, P2Y12 inhibitor monotherapy should be considered after a short-duration DAPT, based on the individual's ischemia and bleeding risks (56). Thus, balancing the risks of ischemia and bleeding when using DAPT has always been a clinical challenge.

This study has several limitations. First, the sample sizes of several RCTs and the number of RCTs are relatively small. Second, the follow-up duration subsequent to PCI varied from in-hospital to one year or longer. Although we performed subgroup analysis in terms of the duration, the total results might also have swayed to some extent. Third, we pooled trials with heterogeneous populations that varied in study design, disease subtype, treatment strategy, and endpoint definition. Fourth, possible adverse drug reactions, loss of tolerability, and discontinuation of treatment are not incorporated into the consideration.

## Conclusion

We suggest that ticagrelor has a comparable efficacy but a higher risk of bleeding compared to clopidogrel for patients with ACS. Clinicians should selectively adopt ticagrelor or clopidogrel according to different PCI strategies, ethnicities, and countries.

## Data Availability Statement

The raw data supporting the conclusions of this article will be made available by the authors, without undue reservation.

## Author Contributions

LL contributed to the conception or design of the study. MS, WC, and LL contributed to the acquisition, analysis, or interpretation of data for the study. MS and WC drafted the manuscript. LL critically revised the manuscript. All authors gave final approval and agree to be accountable for all aspects of work ensuring integrity and accuracy.

## Conflict of Interest

The authors declare that the research was conducted in the absence of any commercial or financial relationships that could be construed as a potential conflict of interest.

## Publisher's Note

All claims expressed in this article are solely those of the authors and do not necessarily represent those of their affiliated organizations, or those of the publisher, the editors and the reviewers. Any product that may be evaluated in this article, or claim that may be made by its manufacturer, is not guaranteed or endorsed by the publisher.
