# Supplementary material for: Comparison of Clinical Outcomes Between Ticagrelor and Clopidogrel in Acute Coronary Syndrome: A Comprehensive Meta-Analysis
Source: Front Cardiovasc Med. 2022 Jan 27;8:818215. doi: 10.3389/fcvm.2021.818215 (PMC8829718; doi:10.3389/fcvm.2021.818215)
Supplement: Supplementary file 1 [file Data_Sheet_1.doc]

**Supplementary material**

**Supplementary Table S1** PRISMA checklist.

**Supplementary Table S2** Full electronic search strategy through April 25, 2021.

**Supplementary Table S3** Detailed characteristics of the included clinical trials.

**Supplementary Table S4** Detailed characteristics of the included observational studies.

**Supplementary Table S5** Definitions of outcomes.

**Supplementary Table S6** Risk of bias assessment according to the ROB-2.

**Supplementary Table S7** Quality assessment of included observational studies.

**Supplementary Table S8** Subgroup analysis according to enrollment time in RCTs.

**Supplementary Table S9** Sensitivity analysis by including high quality RCTs.

**Supplementary Table S10** Sensitivity analysis by only including studies with clinical event committee-adjudicated events in RCTs.

**Supplementary Table S11** Nonparametric trim-and-fill analysis of publication bias.

**Supplementary Table S1. PRISMA checklist.**

| **Section/topic** | **#** | **Checklist item** | **Reported on page #** |
| --- | --- | --- | --- |
| **TITLE** | | |  |
| Title | 1 | Identify the report as a systematic review, meta-analysis, or both. | 1 |
| **ABSTRACT** | | |  |
| Structured summary | 2 | Provide a structured summary including, as applicable: background; objectives; data sources; study eligibility criteria, participants, and interventions; study appraisal and synthesis methods; results; limitations; conclusions and implications of key findings; systematic review registration number. | 2 |
| **INTRODUCTION** | | |  |
| Rationale | 3 | Describe the rationale for the review in the context of what is already known. | 3 |
| Objectives | 4 | Provide an explicit statement of questions being addressed with reference to participants, interventions, comparisons, outcomes, and study design (PICOS). | 3 |
| **METHODS** | | |  |
| Protocol and registration | 5 | Indicate if a review protocol exists, if and where it can be accessed (e.g., Web address), and, if available, provide registration information including registration number. | 3 |
| Eligibility criteria | 6 | Specify study characteristics (e.g., PICOS, length of follow-up) and report characteristics (e.g., years considered, language, publication status) used as criteria for eligibility, giving rationale. | 4 |
| Information sources | 7 | Describe all information sources (e.g., databases with dates of coverage, contact with study authors to identify additional studies) in the search and date last searched. | 3-4 |
| Search | 8 | Present full electronic search strategy for at least one database, including any limits used, such that it could be repeated. | 3-4, Supplementary Table S2 |
| Study selection | 9 | State the process for selecting studies (i.e., screening, eligibility, included in systematic review, and, if applicable, included in the meta-analysis). | 4 |
| Data collection process | 10 | Describe method of data extraction from reports (e.g., piloted forms, independently, in duplicate) and any processes for obtaining and confirming data from investigators. | 4 |
| Data items | 11 | List and define all variables for which data were sought (e.g., PICOS, funding sources) and any assumptions and simplifications made. | 4, Supplementary Table S5 |
| Risk of bias in individual studies | 12 | Describe methods used for assessing risk of bias of individual studies (including specification of whether this was done at the study or outcome level), and how this information is to be used in any data synthesis. | 5 |
| Summary measures | 13 | State the principal summary measures (e.g., risk ratio, difference in means). | 5 |
| Synthesis of results | 14 | Describe the methods of handling data and combining results of studies, if done, including measures of consistency (e.g., I^2^) for each meta-analysis. | 5 |

| **Section/topic** | **#** | **Checklist item** | **Reported on page #** |
| --- | --- | --- | --- |
| Risk of bias across studies | 15 | Specify any assessment of risk of bias that may affect the cumulative evidence (e.g., publication bias, selective reporting within studies). | 5 |
| Additional analyses | 16 | Describe methods of additional analyses (e.g., sensitivity or subgroup analyses, meta-regression), if done, indicating which were pre-specified. | 5 |
| **RESULTS** | | |  |
| Study selection | 17 | Give numbers of studies screened, assessed for eligibility, and included in the review, with reasons for exclusions at each stage, ideally with a flow diagram. | 6, Fig 1 |
| Study characteristics | 18 | For each study, present characteristics for which data were extracted (e.g., study size, PICOS, follow-up period) and provide the citations. | 6, Supplementary Table S3-S4 |
| Risk of bias within studies | 19 | Present data on risk of bias of each study and, if available, any outcome level assessment (see item 12). | Supplementary Table S6- S7 |
| Results of individual studies | 20 | For all outcomes considered (benefits or harms), present, for each study: (a) simple summary data for each intervention group (b) effect estimates and confidence intervals, ideally with a forest plot. | 6-7, Fig 2-3, Table 1-2 |
| Synthesis of results | 21 | Present results of each meta-analysis done, including confidence intervals and measures of consistency. | 6-7, Fig 2-3, Table 1-2 |
| Risk of bias across studies | 22 | Present results of any assessment of risk of bias across studies (see Item 15). | 8, Fig 4, Table 4 |
| Additional analysis | 23 | Give results of additional analyses, if done (e.g., sensitivity or subgroup analyses, meta-regression [see Item 16]). | 7-8, Table 3, Supplementary Table S8-11 |
| **DISCUSSION** | | |  |
| Summary of evidence | 24 | Summarize the main findings including the strength of evidence for each main outcome; consider their relevance to key groups (e.g., healthcare providers, users, and policy makers). | 8 |
| Limitations | 25 | Discuss limitations at study and outcome level (e.g., risk of bias), and at review-level (e.g., incomplete retrieval of identified research, reporting bias). | 11 |
| Conclusions | 26 | Provide a general interpretation of the results in the context of other evidence, and implications for future research. | 11 |
| **FUNDING** | | |  |
| Funding | 27 | Describe sources of funding for the systematic review and other support (e.g., supply of data); role of funders for the systematic review. | 12 |

**Supplementary Table S2** Full electronic search strategy through April 25, 2021.

#1 Clopidogrel [Mesh]

#2 clopidogrel [Title/Abstract]

#3 iscover [Title/Abstract]

#4 pcr 4099 [Title/Abstract]

#5 sr 25989 [Title/Abstract]

#6 sr 25990c [Title/Abstract]

#7 sr25990c [Title/Abstract]

#8 plavix [Title/Abstract]

#9 OR /1-8

#10 Ticagrelor [Mesh]

#11 Brilinta [Title/Abstract]

#12 Brilique [Title/Abstract]

#13 AZD 6140 [Title/Abstract]

#14 AZD6140 [Title/Abstract]

#15 Ticagrelor [Title/Abstract]

#16 OR/10-15

#17 MYOCARDIAL ISCHEMIA [Mesh]

#18 (myocardial OR heart OR cardiac) AND infarct* [Title/Abstract]

#19 coronary AND (thrombosis OR arteriosclerosis OR syndrome* OR acute) [Title/Abstract]

#20 (myocardial OR heart) AND (ischaemi* OR ischemi*) [Title/Abstract]

#21 (coronary OR heart) AND disease* [Title/Abstract]

#22 acute angina [Title/Abstract]

#23 unstable AND angina [Title/Abstract]

#24 ami [Title/Abstract]

#25 chd [Title/Abstract]

#26 ACS [Title/Abstract]

#27 OR/17-26

#28 “Angioplasty, Balloon, Coronary” [Mesh]

#29 “Percutaneous Coronary Intervention” [Mesh]

#30 percutaneous AND coronary AND intervention* [Title/Abstract]

#31 (balloon OR coronary) and angioplast* [Title/Abstract]

#32 coronary and (stent* OR balloon dilation*) [Title/Abstract]

#33 percutaneous coronary [Title/Abstract]

#34 (transluminal or trans-luminal) and coronary [Title/Abstract]

#35 ptca [Title/Abstract]

#36 PCI [Title/Abstract]

#37 invasive management [Title/Abstract]

#38 OR/28-37

#39 #9 AND #16 AND #27 AND #38

**Supplementary Table S3 Detailed characteristics of the included clinical trials.**

| **NO.** | **Study** | **Study**  **Type** | **Country** | **Enrollment** | **Population** | **Ticagrelor group** | | | | | **Clopidogrel group** | | | | | **Follow-up** |
| --- | --- | --- | --- | --- | --- | --- | --- | --- | --- | --- | --- | --- | --- | --- | --- | --- |
|  |  |  |  |  |  | **Sample size** | **Age**  **(year）** | **Male**  **(%)** | **PCI**  **(%)** | **Dosing regimen** | **Sample size** | **Age**  **(year)** | **Male**  **(%)** | **PCI**  **(%)** | **Dosing regimen** |  |
| 1 | Yao, 2017/  Int J Clin Exp Med | RCT | China | 2015.1 - 2016.6 | AMI patients | 60 | 60.4 ± 12.7 | 63.3 | 100 | Loading 180 mg;  maintenance 90 mg once daily | 60 | 59.8 ± 10.8 | 60 | 100 | Loading 600 mg;  maintenance 75 mg once daily | 6 months |
| 2 | Li, 2018/  Clinical cardiology | RCT | China | 2014.1 - 2017.3 | STEMI patients | 161 | 59.8 ± 11.2 | 83.6 | 100 | Loading 180 mg;  maintenance 90 mg twice daily | 281 | 62.8 ± 12.9 | 74.7 | 100 | Loading 600 mg;  maintenance 75 mg once daily | 12 months |
| 3 | Tang, 2016/  J Cardiovasc Pharmacol | RCT | China | 2013.1 - 2015.4 | STEMI patients | 200 | 64.36 ± 11.41 | 71 | 100 | Loading 180 mg;  maintenance 90 mg twice daily | 200 | 64.18 ± 11.09 | 73 | 100 | Loading 600 mg;  maintenance 75 mg once daily | 6 months |
| 4 | Park, 2019/  Circulation | RCT | Korea | 2014.7 - 2017.6 | ACS patients  with or without ST elevation | 400 | 62.5 ± 11.3 | 74.2 | 81.5 | Loading 180 mg;  maintenance 90 mg twice daily | 400 | 62.3 ± 11.5 | 75.5 | 85.5 | Loading 600 mg;  maintenance 75 mg once daily | 12 months |
| 5 | Goto, 2015/  Circulation Journal | RCT | Japan, Taiwan and South Korea | 2011.2-  2012.7 | ACS patients  with or without ST elevation | 401 | 67 ± 12.0 | 76.3 | 84.8 | Loading 180 mg;  maintenance 90 mg twice daily | 400 | 66 ± 11 | 76.7 | 84.5 | Loading 300 mg;  maintenance 75 mg once daily | 12months |
| 6 | Cannon, 2010/  Lancet | RCT | 43 countries | 2006.10-  2009.3 | ACS patients | 6732 | 61.0 (53–69) ^#^ | 74.8 | 76.6 | Loading 180 mg;  maintenance 90 mg twice daily | 6676 | 61.0 (53-70) ^#^ | 74.7 | 77 | Loading 300 mg;  maintenance 75 mg once daily | 12 months |
| 7 | Wang, 2016/  Therapeutics and Clinical Risk Management | RCT | China | 2013.8 - 2014.9 | ACS patients  (65-93 years) | 100 | 79 (76–85) ^#^ | 69 | 75 | Loading 180 mg;  maintenance 90 mg twice daily | 100 | 80  (74–86) ^#^ | 66 | 71 | Loading 300 mg;  maintenance 75 mg once daily | 12 months |
| 8 | Cannon, 2007/  JACC | RCT | 14 countries | 2004.10 - 2005.8 | NSTE-ACS patients | 334 | 64 ± 12.1 | 61 | 42 | Loading 270 mg;  maintenance 90 mg twice daily | 327 | 62 ± 11.0 | 66 | 42 | Loading 300 mg;  maintenance 75 mg once daily | 3 months |
| 9 | Gimbel, 2020/  Lancet | RCT | Netherlands | 2013.6 - 2018.10 | NSTE-ACS patients (aged 70 years or older) | 502 | 77 (73–82) ^#^ | 65 | 48 | Loading 180 mg;  maintenance 90 mg twice daily | 500 | 77  (73–81) ^#^ | 63 | 46 | Loading 300 mg or 600 mg; maintenance 75 mg once daily | 12 months |
| 10 | Ren, 2015/  Herz | Non -RCT | China | NA | NSTEMI patients | 149 | 56 ± 9.2 | 68.3 | 100 | Loading 180 mg;  maintenance 90 mg twice daily | 151 | 55 ± 8.0 | 70.1 | 100 | Loading 300 mg;  maintenance 75 mg once daily | 12 months |

**Note**:  ^#^recorded as median (Q1-Q3); the rest data of age recorded as mean ± standard error.

**Abbreviations**: ACS, acute coronary syndrome; AMI, acute myocardial infarction; NSTE-ACS, non-ST-elevation ACS; STEMI, ST-elevation myocardial infarction; NSTEMI, non-ST-segment elevation myocardial infarction; PCI, percutaneous coronary intervention; RCT, randomized controlled trial; Non RCT, non-randomized controlled trial; NA, not available.

**Supplementary Table S4 Detailed characteristics of the included observational studies.**

| **NO.** | **Study** | **Study type** | **Country** | **Enrollment** | **People** | **Ticagrelor group** | | | | | **Clopidogrel group** | | | | | **Data type** | **Follow-up** |
| --- | --- | --- | --- | --- | --- | --- | --- | --- | --- | --- | --- | --- | --- | --- | --- | --- | --- |
|  |  |  |  |  |  | **Sample size** | **Age**  **(year）** | **Male**  **(%)** | **PCI**  **(%)** | **Dosing regimen** | **Sample size** | **Age**  **(year)** | **Male**  **(%)** | **PCI**  **(%)** | **Dosing regimen** |  |  |
| 1 | *Turgeon, 2020/  JAMA Internal Medicine | Cohort | Canada | 2012.4-2016.3 | ACS patients | 3711 | 61 (54 - 69) ^#^ | 76.9 | 100 | NA | 3711 | 61 (53 - 71) ^#^ | 75.6 | 100 | NA | Propensity score-matched  /multivariable-adjusted | 12 months |
| 2 | *You, 2020/  JAMA | Cohort | America,  South Korean | 2011.9-2019.3 | ACS patients | 31290 | NA | 70.6 | 100 | NA | 31290 | NA | 70.8 | 100 | NA | Propensity score-matched | 12 months |
| 3 | Yudi, 2016/  Internal Medicine Journal | Cohort | Australia | 2009.7-2013.11 | STEMI and NSTEACS patients | 526 | 61.7 ± 11.8 | 78.1 | 100 | NA | 956 | 67.5 ± 12.8 | 72.0 | 100 | NA | Multivariable-adjusted | 1 month |
| 4 | *Wang, 2018/  Chinese Medical Journal | Cohort | China | 2011.10-2014.8 | ACS patients | 779 | 60.54 ± 10.53 | 71.1 | 100 | NA | 1558 | 60.97 ± 10.54 | 71.7 | 100 | NA | Propensity score-matched | 12 months |
| 5 | Sahle´n, 2016/  European Heart Journal | Cohort | Sweden | 2010.1-2013.12 | AMI patients | 11954 | 67 (59 -75) ^#^ | 71.5 | 88.5 | NA | 33119 | 71 (62 - 80) ^#^ | 65.2 | 67.9 | NA | Multivariable-adjusted | 24 months |
| 6 | *Sun, 2019/  Atherosclerosis | Cohort | China | 2014.8-2017.10 | ACS patients | 1833 | 59.86 ± 10.12 | 74.9 | 100 | NA | 1833 | 60.35 ± 10.62 | 74.0 | 100 | NA | Propensity score-matched | 12 months |
| 7 | Völz, 2020/  J Am Heart Assoc | Cohort | Sweden | 2005.1-2015.1 | ACS patients | 2929 | 67.25 ± 11.64 | 72.7 | 100 | NA | 12168 | 67.30 ± 11.48 | 72..4 | 100 | NA | Multivariable-adjusted | 12 months |
| 8 | *Peyracchia, 2019/  American Journal of Cardiovascular Drugs | Cohort | Contries of  Amecia, Asia  and Europe | 2003 -  2016 | ACS patients | 798 | 60.19@ | 82.7 | 100 | NA | 1831 | 60.51@ | 82.2 | 100 | NA | Propensity score-matched | 12 months |
| 9 | *Park, 2016/  International Journal of Cardiology | Cohort | Korea | 2011.11-2015.6 | AMI patients | 1377 | 62.30 ± 12.06 | 77.7 | 100 | Loading 180 mg;  maintenance 90 mg twice daily | 1377 | 62.24 ± 12.53 | 78.9 | 100 | Loading 300-600mg;  maintenance 75 mg once daily | Propensity score-matched | 6 months |

**Supplementary Table S4.** Continued

| **NO.** | **Study** | **Study type** | **Country** | **Enrollment** | **People** | **Ticagrelor group** | | | | | **Clopidogrel group** | | | | | **Data type** | **Follow-up** |
| --- | --- | --- | --- | --- | --- | --- | --- | --- | --- | --- | --- | --- | --- | --- | --- | --- | --- |
|  |  |  |  |  |  | **Sample size** | **Age**  **(year）** | **Male**  **(%)** | **PCI**  **(%)** | **Dosing regimen** | **Sample size** | **Age**  **(year)** | **Male**  **(%)** | **PCI**  **(%)** | **Dosing regimen** |  |  |
| 10 | Olier, 2018/  Heart (british cardiac society) | Cohort | England  and Wales | 2007.1- 2014.12 | STEMI patients | 13 105 | 63 (53 -72) ^#^ | 74.2 | 100 | NA | 58 248 | 64 (54 -75) ^#^ | 73.5 | 100 | NA | Propensity score-matched  /multivariable-adjusted | 12 months |
| 11 | Krishnamurthy, 2019/  Interventional cardiology | Cohort | UK | 2009.1-2011.12  2013.1-2013.12 | STEMI patients | 811 | 63 (19) ^$^ | 72.4 | 100 | NA | 1648 | 65 (21) ^$^ | 71.5 | 100 | NA | Multivariable-  adjusted | 12 months |
| 12 | Kim, 2019/  Journal of Cardiology | Cohort | Korea | 2013-  2014 | AMI patients | 4811 | 57 (50 - 65) ^#^ | 86 | 100 | Loading 180 mg; maintenance 90 mg twice daily | 15459 | 60 (52 - 68) ^#^ | 81 | 100 | NA | Propensity score-matched  /multivariable-adjusted | 12 months |
| 13 | *Choe, 2019/  International Journal of Cardiology | Cohort | Korea | 2011.11-2015.6 | ACS patients | 1203 | 66 (56 -74) ^#^ | 71.7 | 100 | Loading 180 mg;  maintenance 90 mg twice daily | 1203 | 67 (56 -75) ^#^ | 70.2 | 100 | Loading 600 mg;  maintenance 75 mg once daily | Propensity score-matched  /multivariable-adjusted | 468 days |
| 14 | *Chen, 2016/  Journal of the Chinese Medical Association | Cohort | Taiwan | 2013.7-2015.2 | ACS patients | 224 | 63.8 ± 13.3 | 79.9 | 87.1 | NA | 224 | 63.7 ± 13.7 | 79.5 | 74.6 | NA | Propensity score-matched | 12 months |
| 15 | Zocca, 2017/  EuroIntervention | Cohort | Netherlands | 2012.12-2015.8 | ACS patients | 1053 | 63.9 ± 12.1 | 71 | 100 | Loading 180 mg;  maintenance 90 mg twice daily | 1009 | 62.9 ± 11.6 | 69.6 | 100 | Loading 600 mg;  maintenance 75 mg once daily | Propensity score-adjusted | 12 months |
| 16 | Alexopoulos, 2016/  Journal of Thrombosis and Haemostasis | Cohort | Greece | 2012.1-2013.8 | ACS patients | 717 | 60.1 ± 11.4 | 84.9 | 100 | Maintenance 90 mg twice daily | 959 | 65.3 ± 12.5 | 78.3 | 100 | Maintenance 75 mg once daily | Propensity score-adjusted | 12 months |
| 17 | Welsh, 2019/  Canadian Journal of Cardiology | Cohort | 20 countries | 2010.8-2014.7 | STEMI patients | 2188 | NA | 77.9 | 100 | NA | 6500 | NA | 76 | 100 | NA | Propensity score-adjusted | 12 months |
| 18 | Vercellino, 2017/  BMC Cardiovascular Disorders | Case-control | Italy | 2011.2-2013.6 | STEMI patients | 142 | 66 (56 -73) ^#^ | 73.9 | 92.3 | NA | 259 | 67 (56 - 67) ^#^ | 69.9 | 80.7 | NA | Propensity score-adjusted | 12 months |

**Note**: ^*^Subset following propensity-score matching; ^#^recorded as median (Q1-Q3); @recorded as median; ^$^recorded as median (IQR); the rest data of age recorded as mean ± standard error.

**Abbreviations**: ACS, acute coronary syndrome; AMI, acute myocardial infarction; NSTE-ACS, non-ST-elevation ACS; STEMI, ST-elevation myocardial infarction; NSTEMI, non-ST-segment elevation myocardial infarction; PCI, percutaneous coronary intervention; RCT, randomized controlled trial; Non RCT, non-randomized controlled trial; NA, not available.

**Supplementary Table S5** Definitions of outcomes.

| **NO.** | **Study** | **Study**  **type** | **Major adverse cardiovascular events (MACE)** | **Death/**  **Cardiovascular death** | **Myocardial**  **infarction (MI)** | **Stroke** | **Stent**  **thrombosis** | **Bleeding** |
| --- | --- | --- | --- | --- | --- | --- | --- | --- |
| 1 | Yao, 2017 | RCT | The composite of restenosis of target vessel and non-target vessel, thrombosis, recurrent angina, second MI, all-cause death. | NA | NA | NA | NA | Bleeding (BARC  criteria) |
| 2 | Li, 2018 | RCT | The composite of CV death, MI, stroke. | NA | Non-fatal MI was according to the universal definition proposed in 2007. | Non-fatal ischemic stroke was defined as focal loss of neurological function caused by an ischemic event and diagnosed by computer tomography (CT) or magnetic resonance imaging (MRI). | NA | Bleeding (BARC criteria)  minor bleeding: BARC classification 1;  major bleeding: BARC classification 2 or higher. |
| 3 | Tang, 2016 | RCT | The composite of overall death, MI,  unplanned revascularization, and stroke. | NA | Defined according to the guidelines of the European Society of Cardiology. | Defined as a focal loss of neurologic function caused by either an ischemic or a hemorrhagic event. | In accordance with the Academic Research Consortium criteria. | Bleeding (TIMI criteria) |
| 4 | Park, 2019 | RCT | The composite of death from cardiovascular causes, nonfatal MI, or nonfatal stroke. | Death was considered to have a cardiovascular cause unless an unequivocal, no cardiovascular cause could be established. | Defined according to the Third  Universal Definition. | Defined as a focal loss of neurologic function caused by an ischemic or hemorrhagic event, with residual symptoms lasting at least 24 hours or leading to death. | Defined according to the Academic Research Consortium definition. | Bleeding (PLATO-defined) |
| 5 | Goto, 2015 | RCT | The composite of CV death, MI (excluding silent MI), stroke. | Deaths from vascular causes were those resulting from cardiovascular and cerebrovascular events, or any other death for which there was no clearly documented non-vascular cause. | Defined in accordance with the universal definition. | NA | In accordance with the Academic Research Consortium criteria. | Bleeding (PLATO-defined) |
| 6 | Cannon, 2010 | RCT | The composite of death from vascular causes, MI, or stroke. | Deaths from vascular causes were those resulting from cardiovascular and cerebrovascular events, or any other death for which there was no clearly documented non-vascular cause. | Defined in accordance with the universal definition. | NA | In accordance with the Academic Research Consortium criteria. | Bleeding (PLATO-defined) |
| 7 | Wang, 2016 | RCT | The composite of CV death, MI, or stroke. | Deaths from vascular causes were those resulting from cardiovascular and cerebrovascular events, or any other death for which there was no clearly documented non-vascular cause. | Defined in accordance with the universal definition. | NA | In accordance with the Academic Research Consortium criteria. | Bleeding (any bleeding episode),  PLATO major bleeding (life-threatening and others), and  PLATO minor bleeding (requiring medical intervention) |
| 8 | Cannon, 2007 | RCT | The composite of CV death, MI (including silent MI), stroke. | NA | NA | NA | NA | Bleeding (TIMI criteria) |
| 9 | Gimbel, 2020 | RCT | The composite of cardiovascular death,  myocardial infarction,  stroke | NA | Defined according to the third universal definition of myocardial infarction | Defined as an acute new neurological deficit ending in  death or lasting >24 h not due to another readily identifiable cause such as trauma | Defined  according to the Academic Research Consortium | Bleeding (PLATO-defined) |
| 10 | Ren, 2015 | Non-  RCT | The composite of ischemic stroke, mortality, and vascular events. | NA | NA | NA | NA | NA |
| 11 | Turgeon, 2020 | Cohort | The composite of all-cause death, hospitalization with  nonfatal ACS, coronary revascularization excluding planned staged PCI procedures, or stent thrombosis within 365 days after the index hospitalization. | NA | NA | Ischemic stroke (ICD-10codes I63.0 to I63.9 and I64). | NA | Bleeding (the ICD-10 codes) |
| 12 | You, 2020 | Cohort | The composite of cardiovascular mortality, recurrent AMI, and stroke. | Cardiovascular mortality was identified by a death record with at least 1 cardiovascular-related diagnosis (AMI, stroke, sudden cardiac death, or hospitalization for heart failure) in the 30 days before death. | NA | NA | NA | NA |
| 13 | Yudi, 2016 | Cohort | The composite of death, MI and/or TVR. | NA | An increase in creatine kinase or creatine kinase-MB ≥3 times the upper limit of normal; and/or a significant ST-segment change, development of new Q waves in ≥2 contiguous electrocardiographic leads or new left branch bundle block pattern. | NA | NA | In-hospital bleeding was defined as bleeding requiring a transfusion and/or associated with a prolonged hospital stay and/or a drop in hemoglobin >3 g/dL. |
| 14 | Wang, 2018 | Cohort | The composite of all-cause death, MI, ischemia-driven target vessel revascularization, or stroke. | NA | NA | NA | NA | Bleeding (BARC criteria) |
| 15 | Sahle´n, 2016 | Cohort | The composite of death, readmission for MI, or stroke. | NA | Defined by receiving treatment for any of the following ICD codes: I21-I22. | Defined by receiving treatment for any of the following ICD codes: I60-I64. | NA | Defined by receiving treatment for at least 1 of the following: hemorrhagic stroke, gastrointestinal bleeding, anemia-related bleeding or other bleeding |
| 16 | Sun, 2019 | Cohort | The composite of vascular death, MI and stroke. | Vascular death was defined as death from a cardiovascular causes or cerebrovascular causes and any death without another known cause. | The definition of MI was  consistent with the Third Universal Definition of MI. | Defined as rapid loss of neurologic function caused by an ischemic central nervous system event documented by imaging, with residual symptoms lasting at least 24 h from time of onset or resulting in death. | NA | Bleeding was defined as type 2 or higher bleeding according to the BARC criteria,  Major bleeding was defined as BARC type 3, 4 and 5 bleeding. |
| 17 | Völz, 2020 | Cohort | The composite of death, MI, or stroke. | NA | NA | NA | NA | Major bleeding (BARC type 3),  minor bleeding (BARC type 2). |
| 18 | Peyracchia, 2019 | Cohort | A composite and mutually exclusive endpoint  of death and MI. | NA | According to the ESC fourth universal definition of MI, excluding peri-procedural MI. | NA | NA | Major bleedings were defined as BARC type 3–5 bleedings. |
| 19 | Park, 2016 | Cohort | The composite of cardiac death, non-fatal MI, stroke,  and clinically-driven target vessel revascularization (TVR) at 6-month follow-up. | NA | NA | Defined as a medical condition where blood flow to brain was interrupted, because of either ischemia or hemorrhage. | NA | Major bleeding (TIMI)  Minor bleeding (TIMI) |
| 20 | Olier, 2018 | Cohort | The composite of in hospital mortality, reinfarction  and revascularization. | NA | NA | NA | NA | Bleeding complications were defined as a composite of reported gastrointestinal bleed, intracerebral bleed, retroperitoneal hematoma, tamponade, blood or platelet transfusion, or an arterial access site complication requiring intervention. |
| 21 | Krishnamurthy, 2019 | Cohort | NA | NA | NA | NA | NA | Major bleeding (HORIZONS):  defined as intracranial or intraocular bleeding; access site bleeding of diameter of ≥5 cm, or requiring intervention; a reduction in hemoglobin of ≥40 g/L without  an overt source of bleeding, or ≥30 g/L with an overt source of bleeding; re-operation for bleeding and blood transfusion. |
| 22 | Kim, 2019 | Cohort | NA | NA | Defined as I21, I22, and I23 codes. | Defined by a diagnosis between ICD-10-CM codes I60 to I63 and divided into hemorrhagic (I60-62) and ischemic stroke (I63). | NA | Bleeding events was including  intracranial, gastrointestinal, and other bleeding. Intracranial, gastrointestinal, and other bleeding events |
| 23 | Choe, 2019 | Cohort | The composite of cardiac death, nonfatal MI, or stroke. | All-cause death was defined as any case of death intra- or post-procedure; death was considered to be of a cardiac origin, unless a definite non-cardiac cause was established. | Defined as recurrent symptoms with new electro-cardio graphic changes compatible with MI or cardiac marker levels at least twice the upper limit of normal. | Defined as a new, sudden, focal neurological deficit due to a presumed cerebrovascular cause that was not reversible within 24 h and not due to a readily identifiable cause (e.g., tumors or seizures) | NA | bleeding (TIMI criteria.) |
| 24 | Chen, 2016 | Cohort | The composite of death from vascular causes, MI, or stroke. | Death from vascular causes was defined as death from  cardiovascular or cerebrovascular causes, including deaths due  to an unknown cause. | Defined in accordance with the universal definition proposed in 2012. | Defined as the focal loss of neurologic function caused by an ischemic or a hemorrhagic event with residual symptoms lasting at least 24 hours or eventually leading to death. | According to the Academic Research Consortium  criteria. | Major and minor bleeding (PLATO-defined) |
| 25 | Zocca, 2017 | Cohort | The composite of cardiac death, MI or stroke. | NA | According to the Academic Research Consortium (ARC). | A focal loss of neurologic function by an ischemic or hemorrhagic event, with residual symptoms ≥24 hours or leading to death. | Definite or probable stent thrombosis according to ARC. | Major bleeding was any BARC class 3 or 5 bleeding and/or all TIMI major bleedings. |
| 26 | Alexopoulos, 2016 | Cohort | The composite of death, non-fatal MI, urgent revascularization and stroke. | NA | Defined in accordance with the universal definition proposed in 2007. | Defined as focal loss of neurologic function caused by an ischemic or hemorrhagic event, with residual symptoms lasting at least 24 h  or leading to death. | NA | Bleeding (BARC criteria) |
| 27 | Welsh, 2019 | Cohort | The composite of CV death, MI, or stroke. | NA | NA | NA | NA | Major bleeding included severe (fatal, leading to a decrease in hemoglobin of ≥5 g/dl, significant hypotension, requiring surgery, symptomatic intracranial hemorrhage, or requiring transfusion of 4 or more units of red blood cells), and other no severe major (significantly disabling, intraocular bleeding leading to vision loss, or requiring transfusion of 2-3 units of red blood cells).  Minor bleeding was defined as any other bleeding not meeting criteria for major bleed or requiring 1 unit of blood transfused. |
| 28 | Vercellino, 2017 | Case-control | The composite of cardiovascular death, AMI and stroke. | NA | Defined according to the universal definition. | Defined as focal loss of neurologic function caused by ischemic or hemorrhagic events, with symptoms lasting at least 24 h or leading to death. | NA | Bleeding (BARC  criteria) |

**Abbreviations**: RCT, randomized controlled trial; AMI, acute myocardial infarction; PCI, percutaneous coronary intervention.

**Supplementary Table S6** Risk of bias assessment according to the ROB-2.

| **Unique ID** | **D1** | **D2** | **D3** | **D4** | **D5** | **Overall** |
| --- | --- | --- | --- | --- | --- | --- |
| Cannon 2007 | 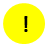 | 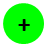 | 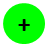 | 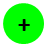 | 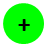 | 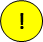 |
| Cannon 2010 | 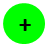 | 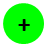 | 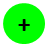 | 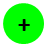 | 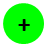 | 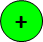 |
| Goto 2015 | 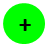 | 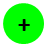 | 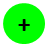 | 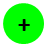 | 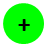 | 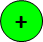 |
| Wang 2016 | 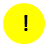 | 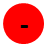 | 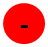 | 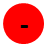 | 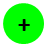 | 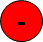 |
| Tang 2016 | 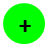 | 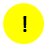 | 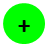 | 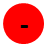 | 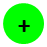 | 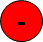 |
| Yao 2017 | 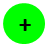 | 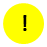 | 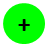 | 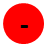 | 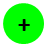 | 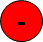 |
| Li 2018 | 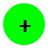 | 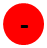 | 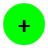 | 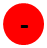 | 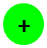 | 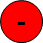 |
| Park 2019 | 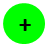 | 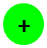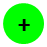 | 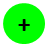 | 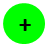 | 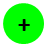 | 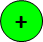 |
| Gimbel 2020 | 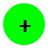 |  | 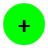 | 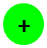 | 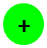 | 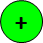 |

Domains: Judgement：


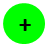
D1: Bias arising from the randomization process Low risk


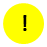
D2: Bias due to deviations from the intended interventions

D3: Bias due to missing outcome data Some concerns


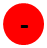
D4: Bias in measurement of the outcome

D5: Bias in selection of the reported result High risk

**Supplementary Table S7** Quality assessment of included observational studies.

| **NO.** | **Study** | **Design** | **Selection** | **Comparability** | **Outcome (cohort)/**  **Exposure (case-control)** | **Study quality** |
| --- | --- | --- | --- | --- | --- | --- |
| 1 | Turgeon, 2020 | cohort | 4 | 2 | 2 | 8 |
| 2 | You, 2020 | cohort | 4 | 2 | 2 | 8 |
| 3 | Yudi, 2016 | cohort | 3 | 2 | 1 | 6 |
| 4 | wang, 2018 | cohort | 3 | 2 | 1 | 6 |
| 5 | Sahle´n, 2016 | cohort | 3 | 2 | 1 | 6 |
| 6 | Sun, 2019 | cohort | 4 | 2 | 3 | 9 |
| 7 | Völz, 2020 | cohort | 3 | 2 | 1 | 6 |
| 8 | Peyracchia, 2019 | cohort | 3 | 2 | 1 | 6 |
| 9 | Park, 2016 | cohort | 3 | 2 | 0 | 5 |
| 10 | Olier, 2018 | cohort | 3 | 2 | 2 | 7 |
| 11 | Krishnamurthy, 2019 | cohort | 4 | 2 | 2 | 8 |
| 12 | Kim, 2019 | cohort | 4 | 2 | 2 | 8 |
| 13 | Choe, 2019 | cohort | 4 | 2 | 3 | 9 |
| 14 | Chen, 2016 | cohort | 3 | 2 | 1 | 6 |
| 15 | zocca, 2017 | cohort | 3 | 2 | 2 | 7 |
| 16 | Alexopoulos, 2016 | cohort | 3 | 2 | 3 | 8 |
| 17 | Welsh, 2019 | cohort | 3 | 2 | 2 | 6 |
| 18 | Vercellino, 2017 | case-control study | 3 | 2 | 3 | 8 |

**Notes:** Quality of observational studies assessed by the Newcastle-Ottawa Scale, with maximum score 9.

**Supplementary Table S8** Subgroup analysis according to enrollment time in RCTs.


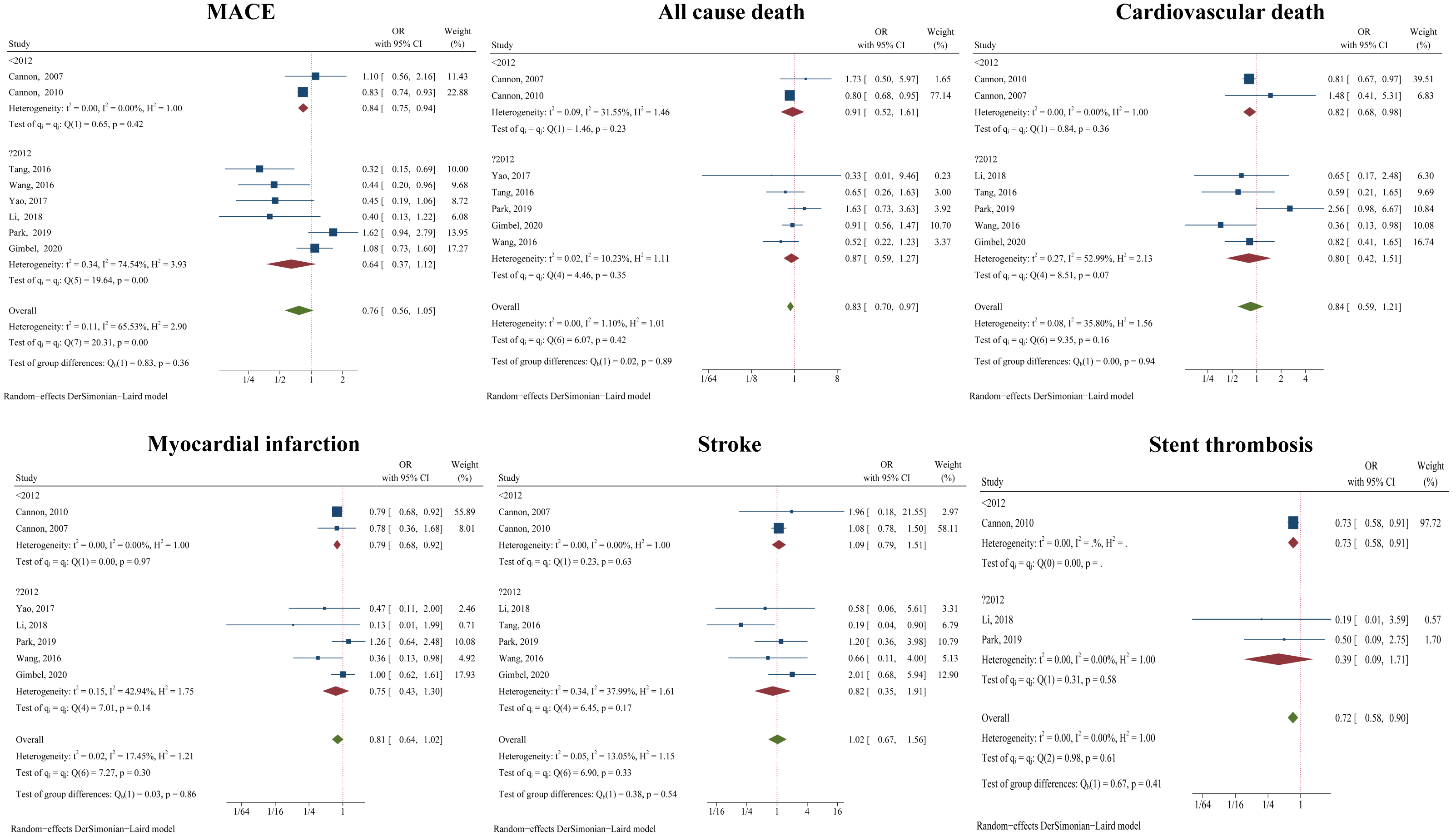


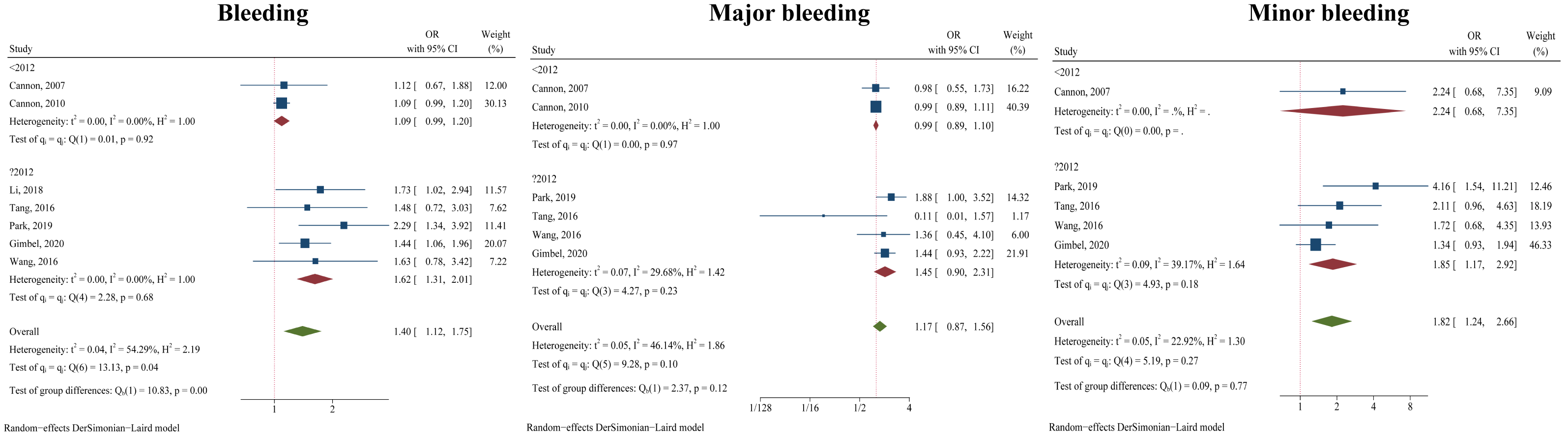


**Supplementary Table S9** Sensitivity analysis by including high quality RCTs.


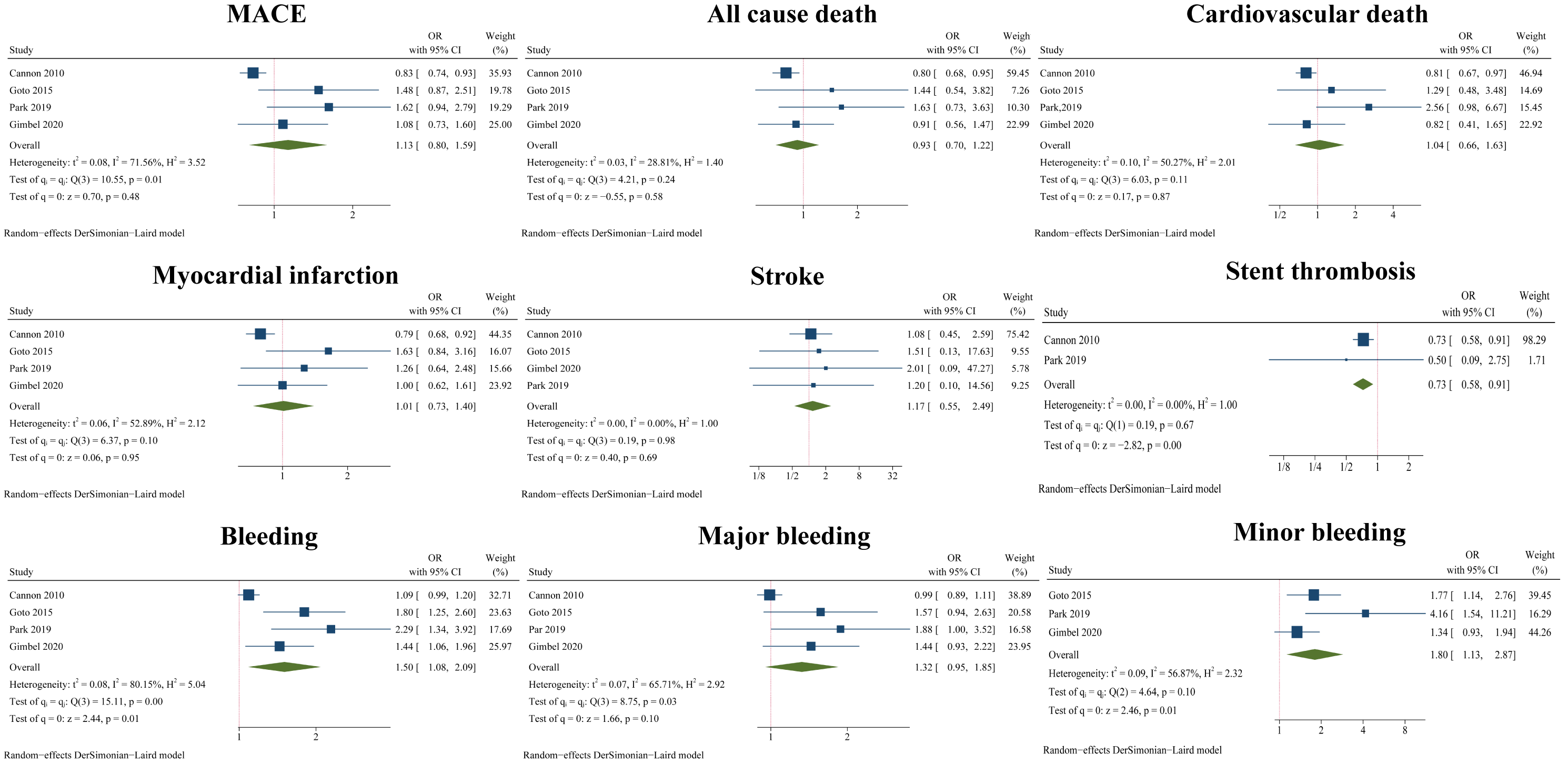


**Supplementary Table S10** Sensitivity analysis by only including studies with clinical event committee-adjudicated events in RCTs.


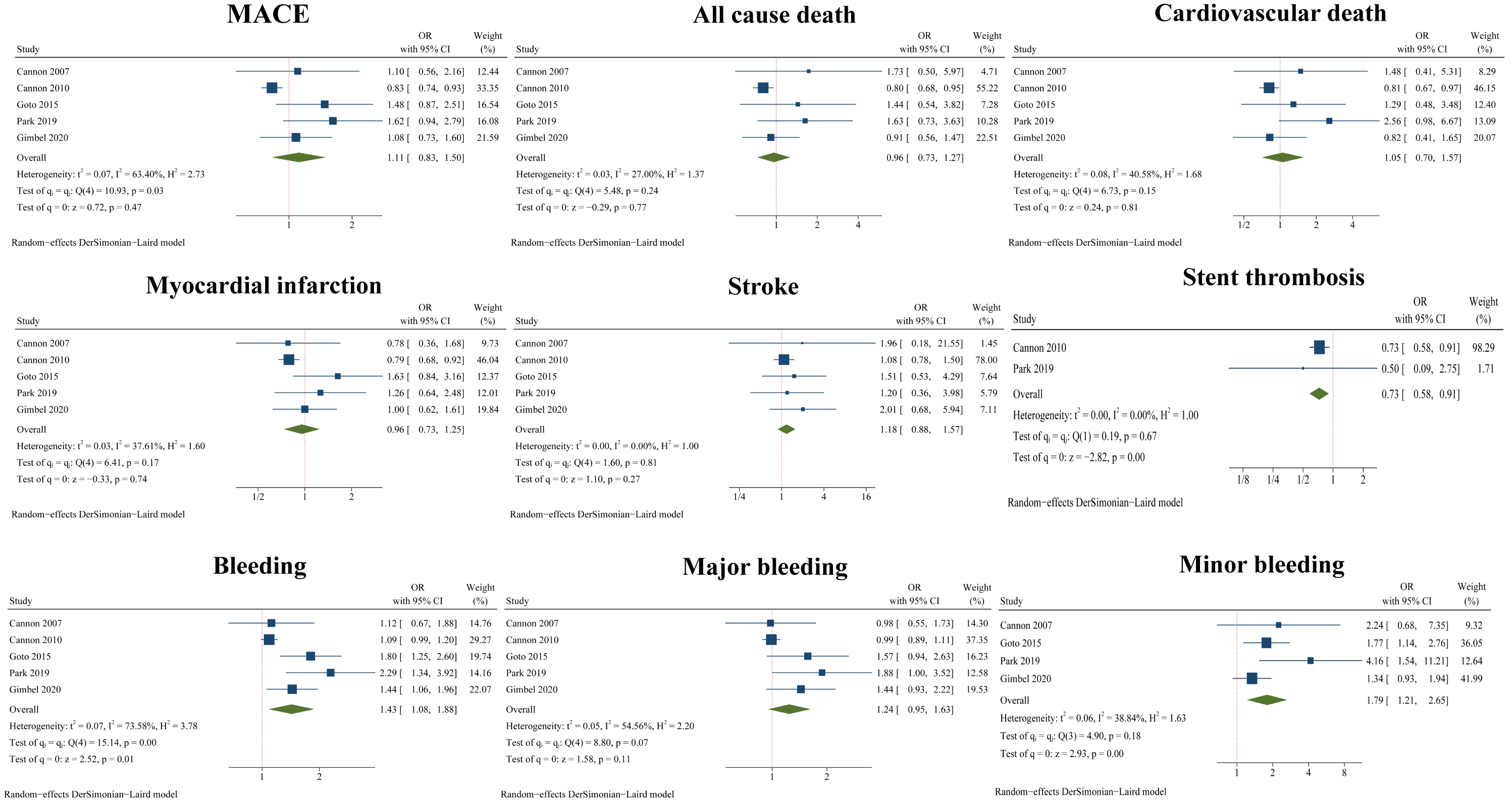


**Supplementary Table S11** Nonparametric trim-and-fill analysis of publication bias.

**A**．Nonparametric trim-and-fill analysis of publication bias in PA group and clinical trials.

| **Outcomes** | **studies** | **number** | **OR** | **95% CI** |
| --- | --- | --- | --- | --- |
| MACE |  |  |  |  |
|  | observed | 23 | 0.856 | 0.755, 0.970 |
|  | observed + imputed | 23+5 | 0.939 | 0.824, 1.069 |
| MI |  |  |  |  |
|  | observed | 20 | 0.995 | 0.944, 1.050 |
|  | observed + imputed | 20+2 | 0.999 | 0.947, 1.054 |
| All-cause death |  |  |  |  |
|  | observed | 21 | 0.845 | 0.738, 0.968 |
|  | observed + imputed | 21+0 | 0.845 | 0.738, 0.968 |
| CV death |  |  |  |  |
|  | observed | 14 | 0.768 | 0.615, 0.958 |
|  | observed + imputed | 14+1 | 0.776 | 0.623, 0.966 |
| Stoke |  |  |  |  |
|  | observed | 19 | 0.907 | 0.744, 1.106 |
|  | observed + imputed | 19+0 | 0.907 | 0.744, 1.106 |
| Stent thrombosis |  |  |  |  |
|  | observed | 10 | 0.83 | 0.689, 1.001 |
|  | observed + imputed | 10+0 | 0.83 | 0.689, 1.001 |
| Bleeding |  |  |  |  |
|  | observed | 15 | 1.435 | 1.207, 1.706 |
|  | observed + imputed | 15+6 | 1.196 | 1.011, 1.414 |
| Major bleeding |  |  |  |  |
|  | observed | 15 | 1.249 | 1.016, 1.535 |
|  | observed + imputed | 15+0 | 1.249 | 1.016, 1.535 |
| Minor bleeding |  |  |  |  |
|  | observed | 10 | 1.633 | 1.429, 1.866 |
|  | observed + imputed | 10+4 | 1.575 | 1.385, 1.791 |

**B**．Nonparametric trim-and-fill analysis of publication bias in MA group and clinical trials.

| **Outcomes** | **studies** | **number** | **OR** | **95% CI** |
| --- | --- | --- | --- | --- |
| MACE |  |  |  |  |
|  | observed | 16 | 0.922 | 0.803, 1.059 |
|  | observed + imputed | 16+2 | 0.961 | 0.832, 1.109 |
| MI |  |  |  |  |
|  | observed | 13 | 0.947 | 0.882, 1.017 |
|  | observed + imputed | 13+1 | 0.947 | 0.882, 1.017 |
| All-cause death |  |  |  |  |
|  | observed | 16 | 0.914 | 0.799, 1.047 |
|  | observed + imputed | 16+0 | 0.914 | 0.799, 1.047 |
| Stoke |  |  |  |  |
|  | observed | 12 | 0.888 | 0.699, 1.127 |
|  | observed + imputed | 12+1 | 0.858 | 0.673, 1.094 |
| Bleeding |  |  |  |  |
|  | observed | 14 | 1.294 | 1.104, 1.516 |
|  | observed + imputed | 14+4 | 1.131 | 0.962, 1.329 |
